# Determinants of wasting among children aged 6–59 months in Wonago woreda, south Ethiopia. A facility-based unmatched case-control study

**DOI:** 10.1371/journal.pone.0269380

**Published:** 2022-06-09

**Authors:** Endashaw Habtamu, Desalegn Chilo, Defaru Desalegn

**Affiliations:** 1 Department of Psychiatry, College of Health Sciences, Dilla University, Dilla, Ethiopia; 2 Department of Pharmacy, College of Health Sciences, Mettu University, Mettu, Ethiopia; 3 Department of Psychiatry, College of Health Sciences, Mettu University, Mettu, Ethiopia; Indian Institute of Technology Kanpur, INDIA

## Abstract

**Background:**

Wasting continued to threaten the lives of 52 million (7.7%) under-five children globally. Sub-Saharan Africa accounts for one-third of all wasted children globally, and Ethiopia is among the countries with the highest magnitude of Wasting in the region. Despite, the little decrement in the prevalence of other forms of malnutrition (stunting and underweight), the burden of wasting remains the same in the country. Gedeo zone is among those with a high prevalence of under-five wasting.

**Objective:**

To identify determinants of wasting among children aged 6–59 months in Wonago Woreda, 2018.

**Methods:**

A facility-based unmatched case-control study was conducted from May 11 to July 21/2018. A total of 356 (119 cases and 237 controls) mothers/caregivers of under-five children who visited the Wonago woreda public health facilities were included in the study using systematic random sampling. Data were collected using a structured questionnaire and anthropometric measurement. Descriptive analysis was used to describe data. Binary logistic regression was used to identify determinants of wasting among children aged 6–59 months. Variables with p-value < 0.25 in bi-variate analysis entered to multivariate analysis. Those variables with a p-value less than 0.05 during the multivariate regression were considered significant.

**Results:**

Determinants which found to have an association with wasting in this study were; maternal illiteracy [AOR = 2.48, 95% CI (1.11, 5.53)] family size <3 [AOR = 0.16, 95% CI (0.05, 0.50)] wealth index [AOR = 2.41, 95% CI (1.07, 5.46)] exclusive breastfeeding in the first 6 months [AOR = 2.71, 95% CI (1.15, 6.40)] dietary diversity [AOR = 5.52, 95% CI (2.06, 14.76)] and children been sick in the last 2 weeks [AOR = 4.36, 95% CI (2.21, 8.61)].

**Conclusion and recommendations:**

Determinants identified were maternal education, family size, wealth index, and exclusive breastfeeding, dietary diversity, and morbidity history of a child in the last 2 weeks. To reduce childhood wasting, due emphasis should be given to empowering women and improving the knowledge and practice of parents on appropriate infant and young child-caring practices.

## Introduction

Malnutrition is an abnormal physiological condition caused by deficiencies, excesses, or imbalances in energy, protein, and/or other nutrients, expressed in the form of over nutrition (obesity) or under-nutrition [1]. Child under-nutrition is a major public health problem, especially in many low-income and middle-income countries [2]. Child malnutrition is responsible for approximately 45 percent of under-five child mortality due to common illnesses, with the majority of deaths occurring in moderately malnourished children [3]. Poor nutrition is linked to suboptimal brain development, which has a negative impact on adult cognitive development, educational performance, and economic productivity [4].

When a child’s nutritional status deteriorates in a relatively short period of time, his or her weight drops to such a low level that they are at risk of dying, the child is said to have acute malnutrition, which is manifested by wasting and being underweight [5].

Wasting refers to a child who is too thin for his/her height and occurs when there is recent rapid weight loss or the failure to gain weight [6]. According to the World Health Organization (WHO) in 2009 child growth standards and identification of severe acute malnutrition in infants and children, wasted children have a weight-for-height ratio of less than -3 SD and/or a MUAC of less than 12.5 cm [7].

Globally, child waste remains a critical issue; its consequences are long-lasting and extend beyond childhood, for example, causing wasted children to have weakened immunity, be vulnerable to long-term developmental delays, and face an increased risk of death [8].

Globally, an estimated of 7.7% and 2.5% of under-five children are wasted and severely wasted, respectively [8]. According to the 2015 Millennium development goal (MDG) report, Sub-Saharan Africa (SSA) accounts for one-third of all wasted children globally, highlighting that wasting still remains a major health problem for children under 5 years in the sub-region, thus showing the need for urgent intervention [9]. Ethiopia is among countries with the highest magnitude of wasting in Sub-Saharan African countries [10].

Being a caretaker living alone [11], poor socioeconomic status and parental illiteracy [12], being in the age group of less than 2 years, birth spacing less than 2 years [13], having large family sizes [14], children aged 12–23 months [15], nonexclusive breastfeeding [16], and suboptimal frequency of complementary feeding [17] are risk factors associated with child wasting. A study was done in the Tigray region also mentioned that children from households who were used for unprotected sources of water were 3.5 times more likely to be wasted compared to protecting sources [18].

Furthermore, cases of high wasting continue to be reported in Southern Nations, Nationalities, and Peoples’ Region (SNNPR) of Ethiopia including: Dilla Zuria, Wonago, Kochore, Bule, and Yirga Cheffe woredas of Gedeo zone, hence they are ranked under priority one for hot spot classification of areas with high malnutrition and food insecurity [19].

In majority of the study, only a few factors like sociodemographic characteristics, breast milk feeding history and maternal and child health service utilization history were repeatedly studied with the same study design and uniform insight across the studies. Variable such as wealth index of the family, families access to variety of food items, the child’s exposure to diverse food groups (dietary diversity score) as well as maternal knowledge, attitude and practices towards adequate child feeding practice were not studied in majority of the studies although, these factors were frequently reported to be predictors of child nutritional status in several researches conducted in other abroad countries. Therefore, this study was aimed to fulfill the above mentioned knowledge gaps and investigate the major determinants of wasting among children aged 6–59 months in the Wonago woreda Gedeo zone, Southern Ethiopia since there are different factors that predispose children to the risk of wasting.

## Methods and materials

### Study design

An Institutional based unmatched case-control study was conducted from May11- July 21, 2018.

### Study setting

The study was conducted in Wonago Woreda, which is one of the 6 Woredas in the Gedeo zone, SNNPRS (Southern Nations, Nationalities, and Peoples Regional State), Ethiopia. The Woreda is found 377 km far away from the capital city, Addis Ababa in the southern direction and 102 km from the region capital Hawassa. The Woreda has 21 kebeles (17 rural and 4 urban) and hosts a total population of 156,480 within 30,442 households. The majority of the population is Gedeo ethnic group. Six health centers, 20 health posts, and 2 private clinics provide the overall health care service in the Woreda. The total number of under-five children in the year 2016 was 4,758 [20].

### Population

All children aged 6–59 months who lived in Wonago woreda where source population and all children aged 6–59 months and who visited health care facilities in Wonago Woreda for varies health care services during the study period were studied population.

### Inclusion and exclusion criteria

#### Inclusion criteria

All children who are a permanent resident or who have lived at least 6 months in wonago woreda aged 6–59 months who visited the health facilities and who have wasted (MUAC<12.5 cm), with their caregivers/mothers who were given informed consent enrolled into the study as cases. Controls included children aged 6–59 months, whose MUAC >12.5 cm and attend health facilities with their mothers/caregivers, who were given informed consent enrolled into the study as controls.

#### Exclusion criteria

Children with physical deformities (children born without hands due to congenital deformities, wounded, and burned hands), children in critical health conditions which made anthropometric measurements inconvenient, and mothers/caregivers who are seriously ill and unable to respond to the questions was excluded from the study.

#### Sample size determination

The sample size was calculated by using Epi-Info version 7 Statistical software by using two population proportions formula with the following assumptions: Squeeze out of first breast milk was taken with proportion of who didn’t squeeze out of first breast milk of the controls to be 53.92% and of the cases 70.1%, 95% confidence interval, 80% power of the study, control to case ratio of 2:1 to detect an odds’ ratio of 2.00 with a 5% of non-response rate [21]. Thus, the sample size required for the study was 366 (122 cases and 244 controls).

#### Sampling procedure

**Fig 1:**
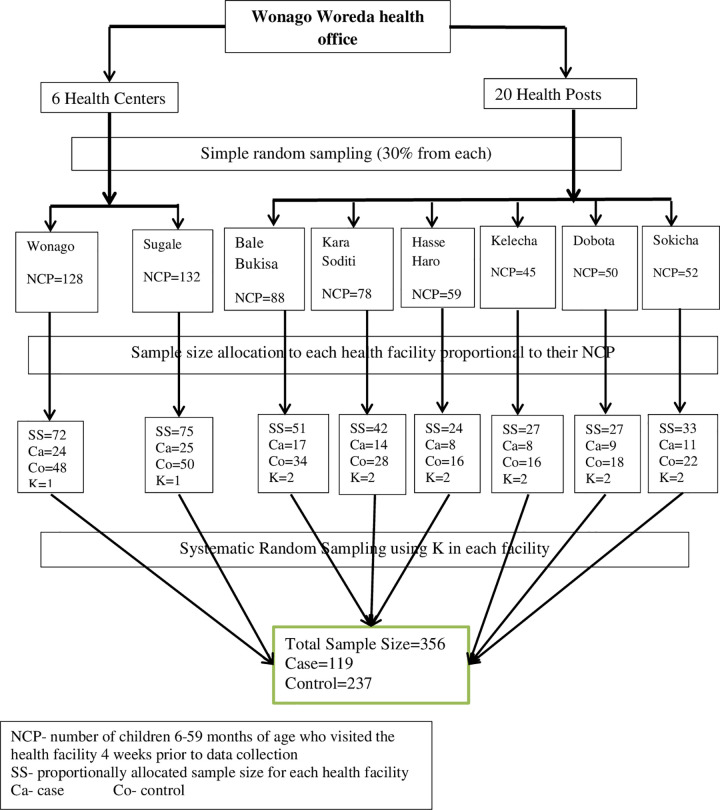
Schematic presentation of sampling procedure for the study on determinants of acute malnutrition among children aged 6–59 months in study area, 2018.

The systematic random sampling technique was applied to select mothers of children aged 6–59 months visiting Wonago public health facilities (health center and health post) for interviews during the study period. The sampling procedure, in general, was as follows: first, the last 4 weeks data of children aged 6–59 months in both public health facilities were taken for the purpose of sample allocation and to determine their sharing’s (Fig 1).

#### Data collection instruments and procedures

The data were collected using a structured questionnaire via face-to-face interviews and anthropometric measurements. Data were collected by structured questionnaire from all eligible children’s mothers/caregivers using a face-to-face interview with 10 Data collectors (4 nurses and 6 health extension workers) and 2 supervisors who have bachelor degrees in public health. It was done under close supervision of the assigned supervisors and principal investigator. A structured interviewer-administered questionnaire was adopted after a detailed review of different pieces of literature and was used to collect data related to the objectives of this study. The questionnaire covered a range of topics including socioeconomic and demographic factors, child characteristics and child-caring practices, maternal characteristics, and environmental health conditions.

The anthropometric data were collected using the procedure set by the WHO (2009) for taking anthropometric measurements. In order to ensure the target population, the age of children was determined before taking anthropometric data. To establish the birth period a local event was used and the mothers or caregivers were asked whether the child was born before or after certain major events until a fairly accurate age was pinpointed. For anthropometric measurement, mid-upper arm circumference (MUAC) was used. MUAC was measured by using a tape meter based on the WHO standardized procedures. Finally, the obtained data of the child were categorized in the wasted and not wasted group according to WHO standard criteria. Edema was checked and determined as per the standard set by WHO, because children with edema were severely malnourished.

To identify the previous sickness of children, mothers were asked about any occurrence of illness during the past two weeks and the vaccination status of children was also checked by observing immunization cards and vaccination scars. Once a case was found and his or her caregiver interviewed, two controls meeting the criteria were selected, and their caregivers were interviewed.

#### Data quality management

The questionnaire was first prepared in English then translates into Amharic and Gede-ufa and back-translated into English by independent translators to check its consistency. Consensus on the compatibility of forward and backward translation was assured before the actual data collection activities.

Data collectors and supervisors were trained for two days by the principal investigator before the actual study commenced on the objectives of the study, Anthropometric measurements, how to interview, and how to handle the questions asked by study subjects. As part of the training, the data collection tool was pre-tested in 5% of the sample size at Dilla zuria wereda (adjacent to the study area) before the actual data collection time to check the extent to which questions understand by the interviewee and to identify areas for modification and correction. Based on the pretest, some chronological arrangements were made. The principal investigator and supervisors checked the completeness and consistency of collecting data on a daily basis, and necessary feedback was given to the data collectors.

Before data entry, in order to make data processing easier, a code was given to each questioner. And data entry format was prepared in Epi Data software according to the pre-coded questioner. To reduce some errors during data entry, a check file was developed (to detect and refuse some data entry mistakes). Before conducting analysis in SPSS software, data cleaning was done to check the presence of outliers, to check consistency, and to verify the skip pattern was followed. In addition, exploratory data analysis was carried out to check the levels of missing values.

### Statistical analysis

The collected data was first coded and entered into a computer using EPI data software version 3.1 and exported to SPSS version 20 statistical software for statistical analysis. Descriptive statistics like frequencies or proportions were presented by tables and figures. In order to investigate determinants of wasting, both bivariate and multivariate analysis was used. Those variables’ p-value of less than or equal to 0.25 in the bivariate was selected as candidate variables for multivariable logistic regression analysis. Multicollinearity was checked by variance inflation factor (VIF). Statistical significance was declared at P-value less than 0.05. Significance of association of the variables was described using AOR with 95% confidence interval.

### Operational definition

Wasting: nutritional deficiency state of recent onset related to sudden food deprivation or malabsorption utilization of nutrients which results in weight loss, MUAC < 12.5 cm from the WHO standard value [7].

Household dietary diversity: dietary diversity is defined as the diversity of plants, animals, and other organisms used as food, covering the genetic resources within species, between species, and provided by ecosystems [22].

Poor dietary diversity: Children who have < 3 food groups.

Good dietary diversity: Children who have > 4 food groups.

Handwashing practice: mean score for all constructs was computed and dichotomized into positive and negative. If the mother scored below the mean, she would be labeled as having poor handwashing practice [23].

Sources of drinking water: according to WHO [24].

Improved water source: piped water to premises, Public taps or standpipes, tube wells or boreholes, protected dug wells, protected springs, Rainwater collection.

Unimproved water source: surface water, unprotected dug well, unprotected spring, Cart with small tank/drum, Surface water, bottled water.

### Ethical consideration

Ethical clearance was obtained from the Ethical Review Board of Dilla University College of Medicine and Health Science, School of Public and Reproductive Health. Then by explaining the importance and the intention of the study, an official letter of co-operation was taken from the Wonago health office. Written informed consent from parents/caretakers of the study subject was obtained after the objective of the study was explained to them. Privacy and confidentiality of collecting information were insured at all levels. Participants were assured that if they wish to refuse to participate, their care or dignity has not compromised in any way. Participants also were informed that there is no expectation of additional treatment or any associated benefits and risks for them participating in the study.

## Results

A total of 356 children between the ages of 6–59 months participated in this study with a response rate of 97%. Study participants were selected in accordance with for every one case selected systematically two children were selected as control, i.e. case to control the ratio of 1:2. Thus, data analysis was done based on data collected from 119 cases and 237 controls.

### Sociodemographic and economic characteristics of the study participants

More than half of the children included in the study were male in sex 190 (53.4%). A majority, 255 (71.6%) of the children, were in the age range of under 12 months. About 345 (96.9%) of the children’s families were living together, married, at the time of the data collection. The majority 296 (83.5%) of the participants were rural inhabitants. And 290 (81.5%) children’s families were protestant in their religion and 80.6% of them were Gedeo in ethnicity (**Table 1**).

**Table 1:** Socio-demographic and economic characteristics among children aged 6–59 months in Wonago Woreda, South Ethiopia, November 2018.

Characteristics	Frequency					
	Cases		Controls		Total	
	N	%	N	%	N	%
**Child’s age**						
< 12	90	75.6	165	69.6	255	71.6
12–24	16	13.4	33	13.9	49	13.8
> 24	13	10.9	39	16.5	52	14.6
**Marital status**						
Married	111	93.3	234	98.7	345	96.9
Divorced	5	4.2	2	0.8	7	2.0
Widowed	3	2.5	1	0.4	4	1.1
**Maternal education**						
Illiterate	63	52.9	102	43.0	165	46.3
Literate	56	47.1	135	57.0	191	53.7
**Paternal education**						
No formal education	36	30.3	41	17.3	77	21.6
Formal education	83	69.7	196	82.7	279	78.4
**Maternal occupation**						
House wife	87	73.1	137	57.8	224	62.9
Farmer	19	16.0	23	9.7	42	11.8
Daily laborer/ merchant/employee	13	10.9	77	32.4	90	25.3
**Paternal occupation**						
Farmer	67	56.3	70	29.5	137	38.5
Daily laborer	49	41.2	102	43.0	151	42.4
Merchant	2	1.7	49	20.7	51	14.3
Employee	1	0.8	16	6.8	17	4.8
**Family size**						
< 3	10	8.4	46	19.4	56	15.7
3–4	26	21.8	63	26.6	89	25.0
≥5	83	69.7	128	54	211	59.3
**Wealth index**						
Very poor	58	48.7	61	25.7	119	33.4
Poor	29	24.4	89	37.5	118	33.2
Rich	32	26.9	87	36.8	119	33.4

### Child caring practice characteristics of the study participants

Overall, about 84.3% of the children in the current study were put into breastfeeding immediately after birth. Of all children, around three fourth (72.8%) were breastfed exclusively for the first 6 months after birth (**Table 2**).

**Table 2:** Child caring practice characteristics among children aged 6–59 months in Wonago Woreda, South Ethiopia November 2018.

Characteristics	Frequency					
	Case		Control		Total	
	N	(%)	N	(%)	N	(%)
**Early initiation of breast feeding**						
Yes	82	68.9	218	92.0	300	84.3
No	37	31.1	19	8.0	56	15.7
**Exclusive breast feeding under 6 months**						
Yes	66	55.5	193	81.4	259	72.8
No	53	44.5	44	18.6	97	27.2
**Initiation of complementary feeding**						
<6 months	26	21.8	8	3.4	34	9.6
6–12 months	30	25.2	172	72.6	202	56.7
13–24 months	61	51.3	35	14.8	96	27.0
>24months	2	1.7	22	9.3	24	6.7
**Frequency of complementary feeding**						
≤2meal/day	74	62.2	157	65.8	228	64.6
≥3meal/day	45	37.8	80	34.2	128	35.4
**Dietary diversity**						
Good	8	6.7	83	35.0	91	25.6
Poor	111	93.3	154	65.0	265	74.4

### Child health status and health service utilization characteristics of the study participants

**Fig 2:**
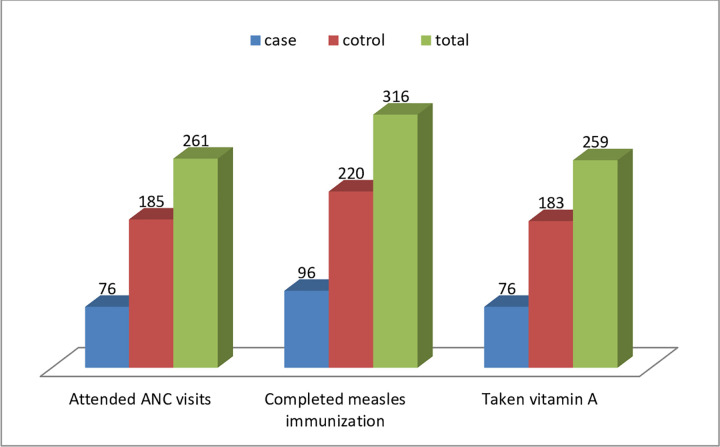
Health service utilization characteristics of children aged 6–59 months in Wonago Woreda, South Ethiopia November 2018.

The majority of study participants 236 (66.3%) both in cases 65 (54.6%) and controls 171 (72.2%) do not take drugs for the intestinal parasites in the past 6-month prior to the date of the survey. Among all 181 (52.0%) children and from control, 153 (64.6%) children have not been sick in the last 2 weeks before study time. In contrast, from case 87 (73.1%) children have been sick in the last 2 weeks before the study period, among them 73.5% of them had the diarrheal disease. Almost three fourth of all mothers 261 (73.3%) both in cases 76 (63.9%) and controls 185 (78.1%) attended ANC visits during their last pregnancy (Fig 2).

### Environmental health and hygiene characteristics of the study participants

Almost all participants 265 (74.4%) of which 186 (78.5%) were from controls and 79 (66.4%) of mothers/caretakers from cases reported that they have access to an improved water source. Conversely, slightly above half 172 (51%) of cases and nearly one in every half 63 (18%) mothers/caretakers from controls reported to have poor handwashing practices. In particular, about 34 (14.7%) and 14 (11.8%) from controls and cases, respectively have reported to lack any form of latrine in their compound or nearby. The majority of the 198 (55.6%) similarly majority in cases do not treat drinking water before consumption, but the majority of respondents 127 (53.6%) among controls are able to treat drinking water before consumption. In addition, around half of all participants 172 (48.3%) and the majority 139 (58.6%) of controls remove solid waste by damping to the ground by composting. Unlike control’s majority, 69 (58.0%) of respondents from cases remove solid waste in an open field.

### Determinants of wasting among children aged 6–59 months in Wonago Woreda, South Ethiopia

In order to investigate determinants of wasting, both bivariate and multivariate analysis was used. Those variables’ p-value of less than or equal to 0.25 in the bivariate was selected as candidate variables for multivariable logistic regression analysis. Variables which significantly associated in bivariate analysis were: Paternal education, Family size, Wealth index, Early initiation of breastfeeding, exclusive breastfeeding in the first 6 months, Children who started complementary feeding at age <6 months, Dietary diversity, Vaccination for measles, De-worming in the last 6 months, Vit A in the last 6 months, Children who have been sick in the last 2 weeks, Source of drinking water, Method of waste disposal, Handwashing practice.

The multivariable logistic regression analysis was used by taking all such factors into account simultaneously, and only the following seven of the most contributing factors remained to be significantly and independently associated with wasting. Determinants that were significantly associated with wasting in this finding were; maternal illiteracy, Family size, Wealth index, Exclusive breastfeeding in the first 6 months, Dietary diversity, Children who have been sick in the last 2 weeks.

Maternal illiteracy was showing a statistically significant association with the outcome variables. Those children whose mother had no formal education had 2.48 times higher risk of wasting as compared to those children whose mother had formal education [AOR = 2.48, 95% CI (1.11, 5.53)]. Family size was shown a statistically significant association with the outcome variables. Those children from family size <3 has 84% less likely to be wasted compared to those who have family size >5 [AOR = 0.16, 95% CI (0.05, 0.50)]. The wealth index is also having a statistically significant association with the outcome variable. Those children from low family wealth index have 2.41 times higher risk of wasting as compared to children from high family wealth index quantile [AOR = 2.41, 95% CI (1.07, 5.46)]. Exclusive breastfeeding in the first 6 months was shown a statistically significant association with the outcome variable. Those children who didn’t have exclusive breastfeeding in the first 6 months were 2.71 times higher risk of wasting as compared to those who have exclusively breastfed in the first 6 months [AOR = 2.71, 95% CI (1.15, 6.40)].

Children with poor dietary diversity were found to be 5.52 times more likely to have wasting than Children with good dietary diversity [AOR = 5.52, 95% CI (2.06, 14.76)]. Children who have been sick in the last 2 weeks preceding data collection were found to be 4.36 times at high risk of developing wasting as compared to those who have not been sick in the last 2 weeks preceding data collection [AOR = 4.36, 95% CI (2.21, 8.61)] (**Table 3**).

**Table 3:** Determinants of wasting among children aged 6–59 months in Wonago Woreda, South Ethiopia November 2018.

Variable	Outcome				
	Case	Control	COR (95% CI)	AOR (95% CI)	P-value
**Maternal education**					
No formal education	63	102	1.48 (0.95, 2.31)	**2.48 (1.11, 5.53)**	0.02
Formal education	56	135	1	1	
**Paternal education**					
No formal education	36	41	2.07 (1.23, 3.47)	2.11 (0.85, 5.21)	0.10
Formal education	83	196	1	1	
**Family size**					
< 3	10	46	0.33 (0.16, 0.70)	**0.16 (0.05, 0.50)**	< 0.01
3–4	26	63	0.63 (0.37, 1.08)	**0.18 (0.07, 0.45)**	< 0.01
≥5	83	128	1	1	
**Wealth index**					
Low	58	61	2.58 (1.50, 4.44)	**2.41 (1.07, 5.46)**	0.03
Middle	29	89	0.88 (0.49, 1.58)	1.79 (0.78, 4.06)	0.16
High	32	87	1	1	
**Early initiation of breast feeding**					
**Yes**	82	218	1	1	
**No**	37	19	5.17 (2.81, 9.51)	1.99 (0.67, 5.84)	0.20
**Exclusive breast feeding under 6 months**					
**Yes**	66	193	1	1	
**No**	53	44	3.52 (2.16, 5.73)	**2.71 (1.05, 6.40)**	0.02
**Dietary diversity**					
**Good**	111	154	1	1	
**Poor**	8	83	7.4 (3.47, 16.07)	**5.52 (2.06, 14.76)**	< 0.01
**Vaccination for measles**					
**Yes**	97	221	1	1	
**No**	22	16	3.13 (1.57, 6.22)	1.59 (0.55, 4.56)	0.38
**De-worming in the last 6 months**					
**Yes**	66	171	1	1	
**No**	53	66	2.08 (1.31, 3.29)	0.66 (0.28, 1.57)	0.35
**Vit A in the last 6 months**					
**Yes**	77	182	1	1	
**No**	42	55	1.80 (1.11, 2.92)	1.85 (0.75, 4.54)	0.17
**Sick child in the last 2 weeks**					
**Yes**	85	96	3.67 (2.28, 5.90)	**4.36 (2.21, 8.61)**	< 0.01
**No**	34	141	1	1	
**Source of drinking water**					
**Improved**	79	186	1	1	
**Unimproved**	40	51	1.84 (1.13, 3.01)	0.63 (0.24, 1.66)	0.35
**Method of waste disposal**					
**Open field**	72	68	2.26 (1.10, 4.64)	1.20 (0.34, 4.30)	0.76
**Composting**	33	139	0.50 (0.24,1.06)	0.34 (0.09, 1.19)	0.09
**Burning**	14	30	1	1	
**Hand washing practice**					
**Poor practice**	19	20	2.06 (1.05, 4.03)	0.81 (0.30, 2.21)	0.68
**Good practice**	100	217	1		

## Discussion

Determinants which significantly associated with wasting in this finding were; educational status of mothers, family size of household, the wealth index of the household, exclusive breastfeeding under 6 months, food security, and dietary diversity.

Among sociodemographic factors in this study, maternal educational status is significantly associated with wasting. Those children whose mother had no formal education had 2.48 times higher risk of wasting as compared to those children whose illiterate mothers [AOR = 2.48, 95% CI (1.11, 5.53)]. In line with this study, a similar study was conducted in Dilla town, Gedeo zone, Children whose mothers are illiterate were 4.18 times more likely to have wasted compared to children whose mothers are literate (AOR = 4.18, CI = (1.36–12.8)) [21].

Similarly, another study conducted in the Oromia region, west Ethiopia; Konso, Southern Ethiopia revealed that those children whose mothers/caregivers are illiterate were more likely to have wasted as compared to those with literate mothers/caregivers [14,25]. This shows that improved maternal education enhances mothers’ knowledge and practice towards child feeding practices, and empowers them to involve in better economic status than their counterparts. Thus, this can be hypothesized as the maternal education may simply be a proxy for child undernutrition factors such as child caring practice and health-seeking behavior which directly affects the children’s nutritional status.

Family size showed a statistically significant association with the outcome variables. Those children from family size <3 has 84% less likely to have wasted compared to those who have family size >5 [AOR = 0.16, 95% CI (0.05, 0.50)]. In line with the finding of this study, a study conducted in the Oromia region, west Ethiopia has large family sizes (AOR = 2.59 (95% CI) (1.34,5.0)) Children from family size greater than or equal to 5 were 2.59 times more at risk to be wasted compared to children from family size less than five [14].

Other similar studies conducted in the Afar region, Northeast Ethiopia and Gobu Sayo Woreda, East Wollega Ethiopia also showed that children from larger family sizes of the household having five and above were more likely to develop wasting as compared to the family size of the household less than five [15,26]. Increased division of available resources in the household results in a nutritional shortfall. This supports the idea that non-nutritional factors should be essential components in the effort to reduce acute malnutrition in Ethiopia. This is due to the fact that mothers who had many Children may not have appropriate child feeding care and nearby mother intimacy.

Another important sociodemographic factor that has a significant effect on the nutritional status of under-five children is the wealth status of the family. Those children from low family wealth index have 2.41 times higher risk of wasting as compared to children from high family wealth index quintile AOR = 2.41, 95% CI (1.07, 5.46). A study conducted in Lalibela Town, North Ethiopia revealed that children living within a household in the highest wealth quintile were 49% less likely to be underweight compared to those children living within a household in the lower wealth quintile AOR = 0.51; (95%CI: 0.28–0.91) [27].

Among child caring practice factors, Exclusive breastfeeding for 6 months was found to be associated with being wasted. Children who were not exclusively breastfed 6 months of age were 2.71 times more likely to have wasted compared to those who were exclusively breastfed 6 months of age [AOR = 2.71, 95% CI (1.05, 6.40)]. In line with this, a study conducted in Enebsie Sarmidr Woreda, North West Ethiopia showed that children who started complementary feeding before 6 months were 5.81 times at more risk of developing wasting as compared to children who were started complementary feeding at 6 months (AOR = 5.81, 95% CI 1.80–18.79) [16].

Other findings that support the finding of this study were reported from studies conducted in Dilla town, Gedeo zone; Oromia region, West Ethiopia also showed that children who didn’t have exclusive breastfeeding were more likely to develop wasting as compared to those who were exclusively breastfeed [14,21]. When complementary foods are started, there is a reduction in breast milk consumption, which can lead to a loss of protective immunity [28]. This causes a higher morbidity when unhygienic foods are used, leading to diarrheal disease. In addition, inadequate weaning practices and poor infant feeding practices lead to low protein and energy intake [29]

Children with Poor dietary diversity were found to be at higher risk of wasting than Children with Good dietary diversity. Mothers who feed their child less than or equal to 3 food groups were more likely to develop wasting as compared to those who feed greater than three food groups. Children with Poor dietary diversity were found to be 5.52 times more likely to have wasting than Children with Good dietary diversity [AOR = 5.52, 95% CI (2.06, 14.76)]. This finding is in line with a study conducted in Karat Town, Southern Ethiopia revealed that Mothers who feed their child less than or equal to 3 food groups were 5.13 times more likely to develop wasting as compared to those who feed greater than three food groups (AOR = 5.13, 95% CI (1.56, 16.84)) [30].

Dietary assessment help determine the risk of deficiency due to low or high intakes of essential nutrients needed for good health it also serves as a proxy for measurement of the nutritional quality of an individual’s diet and in determining whether the child’s diet has the important elements needed for growth or not, In addition, it is a useful indicator for growth as it can serve as a qualitative measure of food consumption and reflect household access to a variety of foods [31].

From child health status factors, the Morbidity of children in the last two weeks showed a statistically significant association with wasting and them, 73.5% of all morbidity status is attributed to diarrheal disease. Children who have been sick in the last 2 weeks preceding data collection were found to be 4.36 times at high risk of developing wasting as compared to those who have not been sick in the last 2 weeks preceding data collection [AOR = 4.36, 95% CI (2.21, 8.61)]. In line with this study conducted by Shashogo Woreda, Southern Ethiopia showed that the presence of diarrhea 2 weeks preceding the survey increased the risk of wasting by 4.13-fold as compared to those who have no diarrhea (AOR = 4.13, 95% CI 1.34–11.47) [17].

A similar finding was obtained in the study conducted in Machakel Woreda, Northwest Ethiopia, where diarrhea increases the risk of wasting nearest to three times [32]. Infections play a major role in the etiology of undernutrition because they result in increased needs and high energy expenditure, lower appetite, nutrient losses due to vomiting, diarrhea, poor digestion, malabsorption and the utilization of nutrients, disruption of metabolic equilibrium and diarrhea is the leading cause of morbidity and mortality of children through dehydration and malnutrition.

## Conclusions and recommendations

In this study, several factors like maternal education, family size, and wealth index, exclusive breastfeeding, early initiation of complementary feeding, dietary diversity, and morbidity history of a child in the last 2 weeks were identified as determinants of wasting. Hence, in order to reduce childhood wasting, due emphasis should be given to empowering women and improving the knowledge and practice of parents on appropriate infant and young child-caring practices.

## Strength and limitations of the study

The strength of the study was that we have examined multiple determinant factors of wasting. As a limitation it is possible that selection bias may occur since controls were selected from institutions, but efforts were made to differentiate cases and controls through clinical assessment (child history and physical examination) and additionally we made two controls for a single case to minimize such type of selection bias. And also recall bias might occur. But, we have tried to minimize it by using data collectors who were experienced, who know the local languages and community practices and they have helped mothers try to recall by local event recalls like local holidays.

## Abbreviations

ANC: Antenatal Care
AOR: Adjusted odds ratio
CBN: Community based nutrition
COR: Crude odds ratio
CSA: Central statistical agency
EDHS: Ethiopian Demographic Health Survey
GDP: Gross domestic product
G.Z.H.B: Gedeo zone health bureau
MAM: Moderate acute malnutrition
MUAC: Mid upper arm circumference
OPD: Outpatient department
PNC: Postnatal Care
PSNP: Productive safety net program
SAM: Sever acute malnutrition
SDG: Sustainable Development Goal
SNNPR: South nation nationality and people region
UNICEF: United Nations Children’s Fund
WFH: Weight for Height
WHO: World Health Organization
